# Long-term quality of life after ETV or ETV with consecutive VP shunt placement in hydrocephalic pediatric patients

**DOI:** 10.1007/s00381-022-05590-9

**Published:** 2022-07-06

**Authors:** Victoria Bogaczyk, Steffen Fleck, Julia Berneiser, Michael Opolka, Marcus Vollmer, Jörg Baldauf, Christin Maria Gasch, Eva Maria Lemke, Ehab El Refaee, Marc Matthes, Holger Hirschfeld, Heinz Lauffer, Michael Gaab, Henry Schroeder, Sascha Marx

**Affiliations:** 1 Department of Neurosurgery, University Medicine Greifswald, Greifswald, Germany; 2 Department of Neurology, University Medicine Greifswald, Greifswald, Germany; 3 Institute of Bioinformatics, University Medicine Greifswald, Greifswald, Germany; 4 Department of Neuropediatrics, University Medicine Greifswald, Greifswald, Germany; 5 Greifswald, Germany; 6 Hannover Institute of Neurosurgery, Hannover, Germany

**Keywords:** Hydrocephalus, Endoscopic third ventriculostomy, Pediatric, Quality of life

## Abstract

**Purpose:**

Endoscopic third ventriculostomy (ETV) and ventriculoperitoneal shunting are well-established treatments of obstructive hydrocephalus (HCP) in adult and pediatric patients. However, there is a lack of data with regard to the quality of life (QoL) of these patients during long-term follow-up

**Methods:**

Inclusion criteria were pediatric patients with endoscopic treatment of hydrocephalus at the University Medicine Greifswald between 1993 and 2016. Patients older than 14 years at present were assessed with the Short Form-12 (SF-12) questionnaire. Patients younger than 14 years of age were assessed with the KINDL-R questionnaire that was completed by their parents. Patients’ values were compared with the scores of a corresponding age-matched group of the healthy population and with patients who received later shunt treatment. Information about comorbidities, current symptoms, and educational level were gained by an additional part. Comparative analysis between patients with ETV success and failure (defined as shunt implantation after ETV) was performed.

**Results:**

A total of 107 patients (53 m, 54f) were included. Fifty-seven/107 patients (53.3%) were considered as ETV success. Mean age at ETV was 6.9 ± 5.9 years. Fifty-four statements of 89 patients that are still alive were gained (response rate 63%). Of these, 49 questionnaires were complete and evaluable (23 m, 26f; mean age 19.8 ± 10.0 years with an average follow-up period of 13.7 ± 7.2 years). Twenty-six/49 patients (53.1%) are considered ETV success. No statistically significant differences could be obtained between patients with ETV success and ETV failure. Patients older 14 years show QoL within normal range, patients younger than 14 years show significantly lower result regarding their environment of peers and social contacts. Patients younger than 6 months at the time of ETV and patients with posthemorrhagic HCP show significantly lower physical QoL. Gait disturbance, fatigue, and seizures are associated with a lower QoL, and educational level is lower than in the normal population.

**Conclusions:**

Patients who underwent ETV in childhood do not have a lower health-related QoL in general. Subsequent insertions of ventriculoperitoneal (vp) shunts do not lower QoL. Certain subgroups of the patients show lower results compared to the healthy population.

**Supplementary Information:**

The online version contains supplementary material available at 10.1007/s00381-022-05590-9.

## Introduction

Hydrocephalic patients have often repeated or multiple surgical procedures during childhood. Repeated interventions and doctor visits do influence mental development of children [1]. Data with regard to the health-related quality of life (QoL) have been gathered [1–9], but only certain groups have been evaluated (e.g., patients with average IQ ⁠[5], shunt-dependent patients only [⁠^1^, ^9^], patients with aqueductal stenosis [6]).

In view of these data, the aim of this study was to analyze various aspects of QoL of patients with HCP who have been treated with ETV during childhood and in case of missing symptom improvement also with subsequent treatment by shunt. We assume that there are differences in QoL, depending on whether only therapy with ETV was performed or whether further surgical treatments were necessary.

## Methods

Since 1993, all ETVs performed in the Department of Neurosurgery, University Medicine Greifswald, have been collected in a prospectively maintained clinical database. We retrospectively analyzed all pediatric patients treated by ETV between 1993 and 2016. There were no other inclusion or exclusion criteria for our database. Incomplete records were allowed for this study and are marked as “lost to follow-up.” Patients had to be < 18 years at time of ETV. Furthermore, the age, etiology, and preoperative symptoms were registered. All other information (complications, postoperative status, education, MRI) were searched on the basis of the files in the medical archive.

The study was approved by the local ethics board (BB063/17). Patient consent was obtained from all participating patients.

ETV success was defined as absence of further permanent cerebrospinal fluid (CSF) diverting procedures as already established in other studies [10–14]. We studied the written reports of our radiologists. MRI flow sequences (IRTSE and CISS) were accomplished for preoperative planning. Also we studied the surgical reports regarding statements about intraoperative detected obstructions of CSF to clearly define the type of HCP.

### Questionnaires

Due to certain age groups within the follow-up survey, 2 different questionnaires were needed.

*Patients younger than 14 years* of age at the time of the last follow-up were assessed with the KINDL-R questionnaire that was completed by their parents. KINDL-R is a generic instrument that generates 6 dimensions. A maximum score of 100 is possible for each dimension and indicates the best health status [15]. The values of the patients were compared with the corresponding (age-matched) standard cohort. This reference group is based on the National Health Interview and Examination Survey for Children and Adolescents (KiGGS) in Germany [16].

*Patients older than 14 years* at the time of the last follow-up were assessed with the 12-item Short-Form Health Survey (SF-12), a self-reported questionnaire.

The SF-12 is also a generic questionnaire that consists of 12 items and comprises two component scores (Physical Component Summary [PCS] and Mental Component Summary [MCS]). It gives information about physical and mental well-being (average value is 50 with a standard deviation of 10) [17]. Higher scores indicate a higher level of well-being. Mean scores and standard deviation of the two sum scores were calculated for the total study group and subgroups. These scores have been compared with the reference scores which were based on a standard cohort for Germany [17].

*General information* about educational level, current employment, and the course of clinical symptoms was gained with additional questions. This was realized with a specially created questionnaire and specific options for every question as well as a free form for other answers if they were not listed.

### Analysis

Statistical analysis of the SF-12 was executed using the associated SPSS syntax file with SPSS version 25 (IBM Corp., Armonk, NY). All other statistical tests have been performed with GraphPad Prism version 5 (San Diego, California). Welsh’s *t*-test and the Mann–Whitney test for unpaired samples have been used.

Significance level was set at *p* < 0.05.

## Results

### Study group characteristics

A total of 107 (53 m, 54 f) patients met the inclusion criteria. The mean patients’ age at the time of ETV was 6.9 ± 5.9 years. ETV was the first hydrocephalus-related neurosurgical procedure in 65 patients (60.7%). The other 42 patients had previous surgeries as vp shunt-insertion (*n* = 19), insertion of extern ventricle drain (*n* = 11), brain tumor surgery (*n* = 9), other neuroendoscopic procedures (aqueductoplasty, cyst resection) (*n* = 2), or microscopic neurosurgical procedure (foramen magnum decompression) (*n* = 1).

Underlying etiologies of hydrocephalus included tumor-related hydrocephalus (*n* = 40), congenital hydrocephalus (*n* = 29), intracerebral hemorrhage (*n* = 22), postinfectious hydrocephalus (*n* = 11), posttraumatic hydrocephalus (*n* = 2), and arachnoid cysts (*n* = 3). Referring to MR images, 103/107 patients showed an obstruction of CSF pathway. Four/107 patients presented with communicating hydrocephalus and ETV was tried as attempt to avoid permanent vp shunting. Fifty-seven/107 patients (53.3%) were considered as ETV success (see Table 1).

**Table 1:** Patients’ characteristics of QoL group and total study group. Statistical significance set at *p* < 0.05. Statistical tests were done with the Mann–Whitney *U* test and chi-square test

Categorical variables		*n* QoL study group (%)	*n* total study group (%)	*p* value
Total number		49	107	
Gender	Male	23 (46.9)	53 (49.5)	
	Female	26 (53.1)	54 (50.5)	
	Ratio m:f	1:1.13	1:1.02	.766
Age at QoL evaluation (years)	Mean ± SD	19.8 ± 10.0		
	< 6	6 (12.2)		
	6–14	8 (16.3)		
	> 14	35 (71.4)		
Age at time of ETV (years)	Mean ± SD	6.1 ± 5.9	6.9 ± 5.9	.433
	< 6	27 (55.1)	53 (49.5)	
	6–14	15 (30.6)	36 (33.6)	
	> 14	7 (14.3)	18 (16.8)	
Order of surgeries	ETV was first surgery	31 (63.3)	65 (60.7)	.764
	Surgeries before ETV	18 (36.7)	42 (39.3)	
Etiologies	Brain tumor	16 (32.7)	40 (37.4)	.560
	Brain abnormality	15 (30.6)	29 (27.1)	
	Posthemorrhagic	14 (28.6)	22 (20.6)	
	Arachnoidal cyst	2 (4.1)	3 (2.8)	
	Postinfectious	2 (4.1)	11 (10.3)	
	Posttraumatic		2 (1.9)	
Postoperative complication	Meningitis	4 (8.2)	4 (3.7)	.547
	CSF leak	6 (12.2)	12 (11.2)	
ETV success	ETV success	26 (53.1)	57 (53.3)	.840
	ETV failure	23 (46.9)	47 (43.9)	
	No information		3 (2.8)	
Follow-up (years) ± SD		13.7 ± 7.2	9.9 ± 7.5	.0009*

**Fig. 1:**
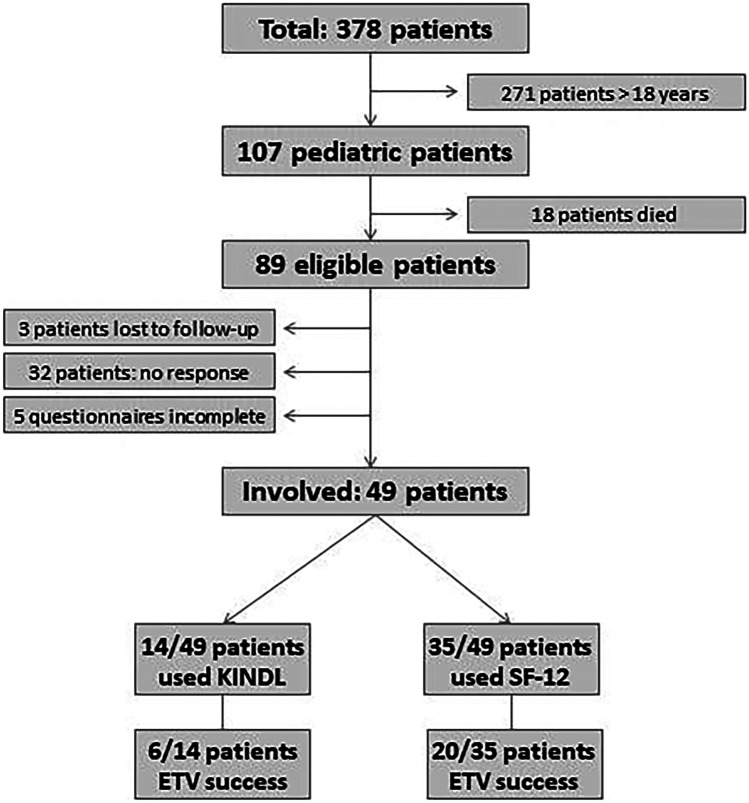
Flow chart about the response and the scheme of evaluation of patients treated with ETV during 1993–2016. SF-12, Short-Form Health Survey 12

Eighty-nine/107 patients were still alive in June 2018. Of these, 3 patients were lost to follow-up immediately after ETV. A total of 54 of the remaining 86 patients agreed to complete our study questionnaires, leading to a response rate of 63% (Fig. 1).

A total of 49 completed questionnaires could be achieved (26f, 23 m) (Fig. 1). The mean patients’ age at the time of assessing the QoL was 19.8 ± 10.0 years. The examined HCP etiologies are as follows: posthemorrhagic HCP (*n* = 14), postinfectious HCP (*n* = 2), brain abnormality (*n* = 15), arachnoidal cyst (*n* = 2), and brain tumor (*n* = 16). The mean patients’ age at the time of ETV was 6.1 ± 5.9 years. Patient’s characteristics are given in Table 1.

Except the length of follow-up time, there are no significant differences in the group of patients with a complete set of questionnaires (*n* = 49) and the total study group (*n* = 107), indicating that the analyzed group is a good representative of the total study group.

### Quality of life

In general, 45 patients (91.8%) described their health status at least as “good.” Only 4 patients (8.2%) reported their health as “average” or “less well.” None of our tests led to statistical significant differences between males and females, that is why we show the following results without gender differentiation.

### Patients younger than 14 years

**Fig. 2:**
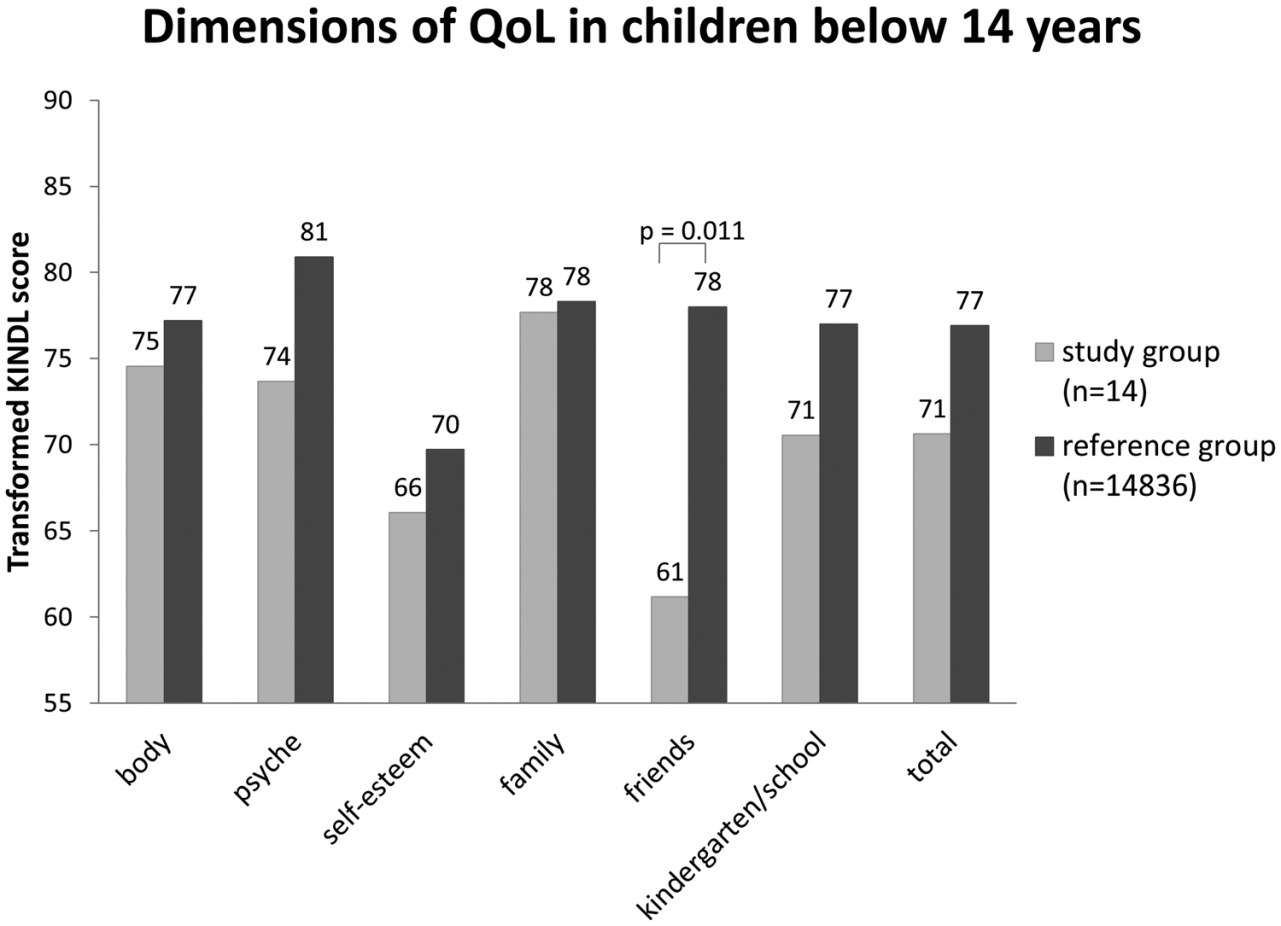
Average health dimensions evaluated with KINDL-R questionnaire in children. QoL, quality of life

Fourteen/49 patients (7f, 7 m) received the KINDL-R questionnaire that was completed by their parents. The mean age was 8.3 years (range 3.0–13.7 years). Eight/14 patients received a vp shunt after ETV. Differences in the QoL between patients with ETV success and failure could not be found (*p* = 0.662). Noteworthy, 7/8 school-aged children attend a special school for handicapped children; only 1 child attends the middle school. The results of the KINDL-R are shown in Fig. 2. All evaluated dimensions are located below the corresponding scores of the reference group [18]. The dimension “friends” shows a statistically significantly lower result compared with the reference group (*p* = 0.011).

### Patients older than 14 years

**Fig. 3:**
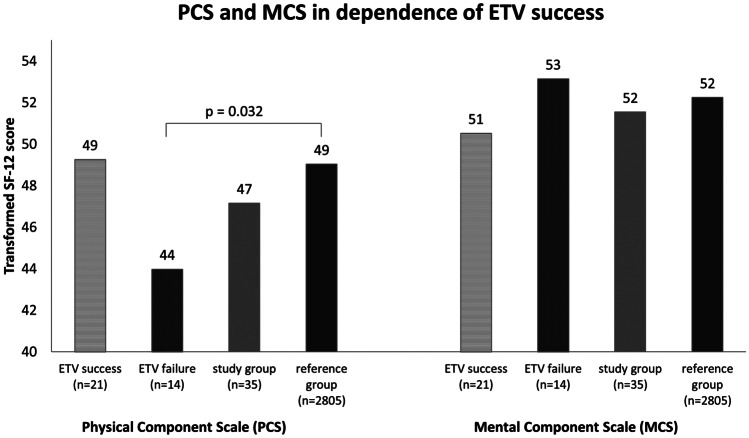
Comparisons between PCS and MCS of patients with ETV success and failure, total study group, and reference group. ETV, endoscopic third ventriculostomy; PCS, physical component summary; MCS, mental component summary

The SF-12 was completed by 35/49 patients (19f, 16 m). The mean age of the survey responders was 24.3 years (range 14.9–39.1 years). The physical dimension of the study group (PCS) reached a score of 47.2 vs. 49.0 of the reference group (*p* = 0.082). The mental well-being of the study group shows a score of 51.5 vs. 52.2 of the reference group (*p* = 0.516) (see overview given in Fig. 3).

Fourteen/35 patients are shunt-dependent at present. Comparison of the ETV failure group with the reference group shows a significantly lower score regarding physical well-being (44.0 vs. 49.0, *p* = 0.032). All other comparisons between the groups, especially regarding MCS, did not show significant differences. Statistical values are given in Table 2; overview is given in Fig. 3.

**Table 2:** Overview about the results of QoL evaluation

SF-12 questionnaire	No. of pat	Score PCS	Score MCS	PCS *p* value compared to study group (*n* = 35)	PCS *p* value compared to reference group (*n* = 2805)	MCS *p* value compared to study group (*n* = 35)	MCS *p* value compared to reference group (*n* = 2805)
Study group	35	47.15	51.54	-	.082	-	.516
Headache	18	49.20	50.10	.374	.643	.680	.982
Gait disturbance	14	38.98	48.90	.038*	.004*	.738	.867
Fatigue	8	42.32	49.04	.310	.047*	.564	.545
Seizures	5	43.82	48.27	.288	.066	.317	.373
ETV success	21	49.26	50.52	.446	.612	.576	.828
ETV failure	14	43.98	53.14	.319	.032*	.465	.194
Posthemorrhagic	7	42.29	55.53	.241	.040*	.219	.321
Brain abnormalities	9	48.63	48.82	.716	.656	.346	.135
Brain tumor	14	48.91	52.39	.535	.624	.691	.280
Younger 6 months	6	40.02	54.29	.117	.034*	.519	.295
Younger 1 year	8	43.84	54.49	.349	.098	.532	.302
1–10 years	14	46.35	50.28	.903	.188	.816	.972
Older than 10 years	13	50.12	51.51	.403	.839	.835	.767
KINDL questionnaire	No. of pat	Score study group		Standard deviation study group	Score reference group (*n* = 14,836)	Standard deviation reference group	*p* value study group vs. reference group
Body	14	74.55		21.30	77.2	27.97	.649
Psyche	14	73.66		17.71	80.9	15.54	.150
Self-esteem	14	66.07		14.44	69.7	18.64	.365
Family	14	77.68		12.91	78.3	18.64	.860
Friends	14	61.16		21.40	78.0	15.54	.011*
Kindergarten/school	14	70.54		19.68	77.0	21.75	.241
Total	14	70.61		13.54	76.9	12.43	.106

**Fig. 4:**
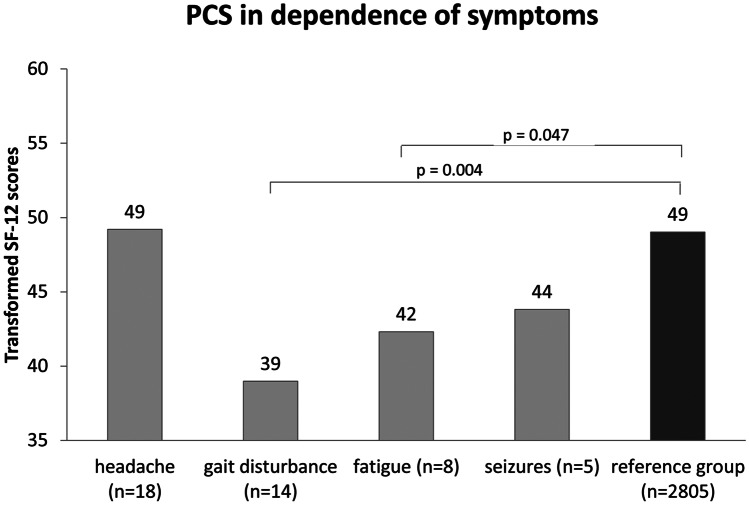
Clinical symptoms and their impact on physical QoL. ETV, endoscopic third ventriculostomy; PCS, physical component summary

Ten/49 patients who have been examined by questionnaire are free of symptoms at current. Thirty-nine/49 patients suffer at least from one health problem:

While nausea (*n* = 2) and vomiting (*n* = 0) hardly play a role, still 18/49 patients (36.7%) suffer regularly from headache. Patients with headache do not show a lower physical or mental QoL compared to healthy controls. Twelve/18 patients with headache are considered ETV success. Gait disturbance is a problem of 14/49 patients (28.6%) showing a significantly lower result regarding physical functioning compared to the reference group (*p* = 0.004). Six/14 patients with gait disturbance are considered ETV success. Mental well-being seems not to be negatively affected. Also 8/49 (16.3%) patients complain about fatigue with significantly lower physical QoL (*p* = 0.047). Five/8 patients with fatigue are considered ETV success. Five/49 patients describe seizures (10.2%). Two/5 patients with seizures are considered ETV success (underlying etiologies were posthemorrhagic HCP, each of the two patients with brain tumor and brain malformation). These five patients showed lower results regarding physical QoL without reaching statistical significance (*p* = 0.066; Fig. 4).

### QoL in association to their age at operation and underlying etiology

Depending on the age at time of ETV, subgroup analysis was executed.

**Fig. 5:**
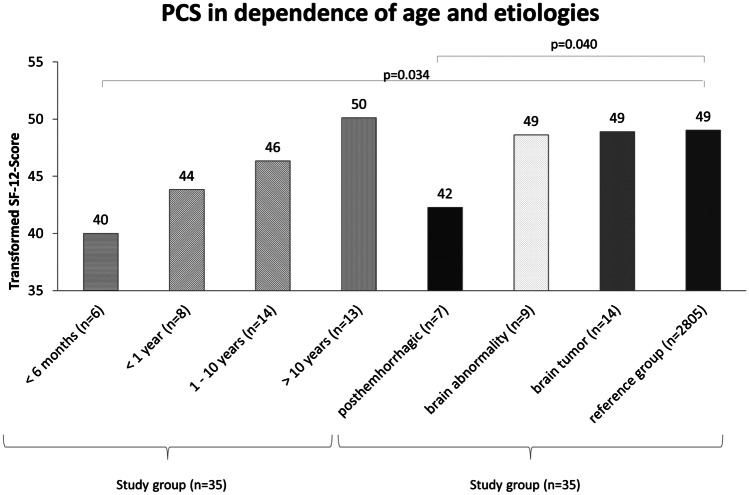
Physical component summary for subgroups compared with the total study group and reference group. PCS, physical component summary

There was no significant difference found in aspects of mental QoL (see Table 1). Differences in physical QoL of subgroups are shown in Fig. 5. In dependence of the patients’ age, PCS increases with higher age at time of ETV. Patients younger than 1 year at time of ETV reached an average PCS of 43.84. Patients between 1 and 10 years reached an average score of 46.35. Patients older than 10 years reached the highest score (average PCS of 50.12). A significant difference in physical QoL was found in patients younger than 6 months (average PCS of 40.02) at time of ETV compared to the reference group (*p* = 0.034).

Considering different subgroups regarding etiologies of HCP, also significant differences in the physical dimension of QoL can be identified (see Fig. 5).

Comparative analysis was possible for the subgroups “posthemorrhagic,” “brain abnormalities,” and “brain tumors.” There was no significant difference found in aspects of mental QoL (see Table 1). Patients with brain abnormalities or brain tumors showed average PCS of 48.63 and 48.91, respectively. A significant difference in physical QoL was found in patients with posthemorrhagic HCP (average PCS of 42.29, *p* = 0.040).

### Education

**Fig. 6:**
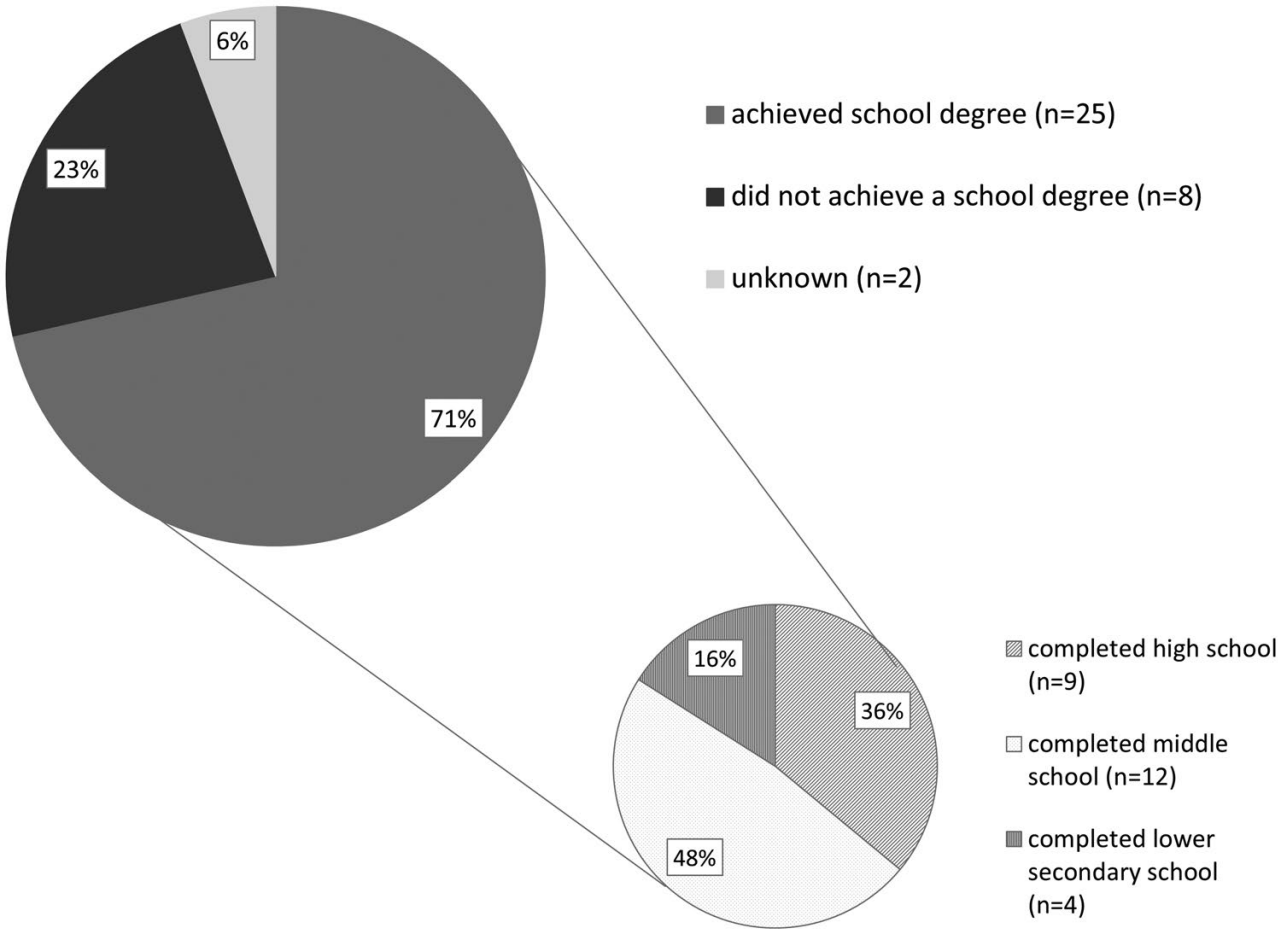
Educational degrees of the study group

In our evaluation, 35/49 patients reported their school education as completed. Twenty-five/35 (71.4%) achieved a school degree. Eight/35 (22.9%) attended a school specialized in education for physically and mentally handicapped children without getting a degree. Two/35 (5.7%) patients did not provide information about their school degree. Nine/25 patients with school degree attended and completed high school. Middle school was attended by 12 patients, and lower secondary school by 4 patients (see Fig. 6).

A total of 29/35 patients (82.9%) are employed at current, of which 8/29 are integrated in a professional environment adjusted to mentally and/or physically handicapped people. The remaining 21/35 patients show a wide spectrum of workspaces.

## Discussion

There are certain generic measure tools including the Health Utility Index, the Pediatric Quality of Life Inventory, and the Child Health Questionnaire as well as condition-specific questionnaires (e.g., Hydrocephalus Outcome Questionnaire) [19–22]. At this date, there is no HCP-specific questionnaire with validated values for German cohorts. We used generic questionnaires which allowed us to compare QoL with the general German population and created a special part with focus on HCP-related health issues.

In general, the results of our study group showed an equal QoL compared to healthy reference groups. Regarding all examined patients older than 14 years, mental well-being was slightly better in shunt-dependent patients whereas physical well-being was better in the ETV success group. The subjectively perceived quality of life cannot always be reconciled with objective criteria. Maybe the physical well-being of patients with a shunt is restricted (e.g., scars tighten, shunt is palpable and disturbing, knowledge of the presence of foreign material), but the reason for these results remains unclear.

Patients younger than 14 years at time of evaluation showed a total score lower than the reference group, but without statistical significance and still within the standard deviation. Kutscher et al. published results showing that adult QoL of shunt-dependent patients with congenital HCP seems to be similar compared to healthy controls [1]. Lindquist et al. reported that QoL was lower in hydrocephalic patients, but without significant statistical differences compared to the reference group [5]. Kulkarni et al. described for certain subgroups (aqueductal stenosis and no other major abnormalities) a comparable QoL with the general population, stating that the overall long-term prognosis is very good [6].

Certain characteristics may cause a lowering of QoL in hydrocephalic patients:

1. Physical impairments. Patients with HCP often suffer from motor and cognitive impairments [3, 7]. Patients with gait disturbances, fatigue, and seizures showed a lower physical QoL in our study group. These results are presumably more caused by etiology than by therapy with shunt or ETV. Kulkarni et al. described headache as one of the most common health issues that patients with HCP have to deal with [3] Prakash et al. reported about 30–42% of patients with vp shunt suffering from headache [23]. These details are comparable to 37% of the patients in our cohort suffering from headache, of which one-third is considered ETV failure.
  Almost 30% of our study group described gait disturbances with a significant reduction of physical QoL. Sixty percent of long-term neuromotoric deficits are described by Hoppe-Hirsch et al. [24]. Besides, fine motor, visual motor, and spatial skills were impaired compared to control groups, and visual or hearing deficits are well-known problems in up to 25% of hydrocephalic patients [25]. According to results of the Fatigue PedsQL accomplished by Sumpter et al. ⁠[7], we also found a statistical significant influence of fatigue regarding physical well-being.
  Epileptic seizures are present in 10% of our cohort leading to a reduced physical QoL. Kulkarni et al. showed an associated reduction of QoL and described epileptic seizures as a well-known problem [3]. Several authors found out that up to 48% of patients with HCP suffer from epileptic seizures [25–28].
2. Social acceptance. In our study group, familiar surrounding was very important and well-functioning for patients. The results were equal compared to healthy controls. Family functioning and family support have also been investigated by other research groups with the result that a poor family surrounding is associated with a decreased child QoL in all dimensions [29]. Our results showed a significantly lower score in regard to the dimension “friends,” which indicates a lack of environment of peers and points out problems in terms of social contacts. Preschool management before peer interaction for improved social integration is a solution presented by Peters et al. [8].
3. Etiology of HCP and age at time of surgery. Patients with HCP due to intracranial hemorrhage and/or patients younger than 6 months at time of ETV showed a significantly lower QoL in our study. Kulkarni et al. also reported lower QoL in patients with intraventricular hemorrhage (IVH). Furthermore, they described a negative influence on QoL due to presence of epileptic seizures, the number of shunt revisions, shunt infections, and longer initial hospital stay [3]. Paulsen et al. focused on QoL measures using SF-36 in 67 shunt-dependent patients with HCP and reported that patients with spina bifida score lower results regarding physical well-being [9]. Kutscher et al. could not find any statistically significant difference between different etiologies, but patients with IVH showed lower scores compared to an aqueduct stenosis group regarding MCS [1]. We did not find any significant differences in relation to MCS.
4. Current shunt dependency. Fourteen/35 patients assessed with SF-12 are shunt-dependent at present. Twenty-one/35 patients who completed the SF-12 are shunt-free. We could not find any statistically significant difference between these two groups in relation to their QoL. However, shunt-dependent patients showed a significantly lower score in physical well-being compared to healthy control groups. In contrast to that, shunt-dependent patients of our study group do not suffer that often from headache or gait disturbances.
  Kulkarni et al. provided a comparison between patients treated with ETV or with shunt during childhood. There were also no striking differences found in regard to QoL [2]. In the more recent prospective, multicenter study with results of triventricular hydrocephalic infants after 5 years (IIHS), it was described that cognitive score measured by Hydrocephalus Outcome Questionnaire showed more favorable results after shunt without reaching statistical significance [6]. As mentioned earlier, Kutscher et al. measured QoL in 31 shunt-dependent patients with congenital HCP using SF-36 questionnaire. Both PCS and MCS showed lower results compared to healthy control groups, whereas only PCS showed a statistically significant lower result. So Kutscher et al. suggested that physical impairment is already a main factor leading to reduced QoL [1].
5. Education. Seventy-one percent (*n* = 25) of our patients completed school and achieved a school degree showing a wide spectrum of workspaces. Compared to all people in Germany between 15 and 25 years, only 3.6% leave school without getting any degree [30].
  Platenkamp et al. reported healthy schooling in 59%, special education in 33%, and no schooling at all in 9% [31]. Describing the late outcome of the surgical treatment of HCP, 40% had dropped out of healthy school curriculum [24]. In addition, Kokkonen presented 82 patients older than 16 years, in whom 46% had healthy intellectual functioning, schooling in 60%, and only 11% with job [32]. Paulsen et al. described that 67% had visit healthy school systems, but stayed behind 1–2 years and needed further help. Furthermore, specific difficulties regarding reading, math skills, or other neuropsychological limitations have been described [33–35]. Educational qualification itself does not appear to be an independent factor influencing QoL, according to results published by Kutscher et al. [1].

### Limitations of the study

An assessment of preoperative QoL was not done. The study group is small and the current analysis is based on a heterogeneous disease spectrum. The effect of an ETV or a shunt itself is difficult to assess in relation to quality of life.

The response rate reflects a probable positive selection bias and might not be wholly representative for this group of patients.

## Conclusions

This long-term follow-up quality of life analysis shows clearly that patients who suffer from HCP and underwent ETV in childhood do not have a lower health-related quality of life than the age-matched group of a healthy population in general. Patients and their families arrange with the disease. We have to improve our instruments to measure QoL. These data about QoL are needed to prove our therapy and to adapt expectations about long-term outcome.

## Supplementary Information

Below is the link to the electronic supplementary material.

Supplementary file1 (DOCX 82 KB)
