# Correction to: Long‑term quality of life after ETV or ETV with consecutive VP shunt placement in hydrocephalic pediatric patients

**DOI:** 10.1007/s00381-022-05657-7

**Published:** 2022-09-06

**Authors:** Victoria Bogaczyk, Steffen Fleck, Julia Berneiser, Michael Opolka, Marcus Vollmer, Jörg Baldauf, Christin Maria Gasch, Eva Maria Lemke, Ehab El Refaee, Marc Matthes, Holger Hirschfeld, Heinz Lauffer, Michael Gaab, Henry W. S. Schroeder, Sascha Marx

**Affiliations:** 1 Department of Neurosurgery, University Medicine Greifswald, 17475 Sauerbruchstraße Greifswald, Germany; 2 Department of Neurology, University Medicine Greifswald, Greifswald, Germany; 3 Institute of Bioinformatics, University Medicine Greifswald, Greifswald, Germany; 4 Department of Neuropediatrics, Greifswald, Germany; 5 University of Greifswald, Greifswald, Germany; 6 Hannover Institute of Neurosurgery, Hannover, Germany

## Correction to: Child's Nervous System https://doi.org/10.1007/s00381-022-05590-9

The original version of this article unfortunately contained an error regarding the order of authors. Given here is the corrected list of authors.

The original article has been corrected.

