# Supplementary material for: Long-term quality of life after ETV or ETV with consecutive VP shunt placement in hydrocephalic pediatric patients
Source: Childs Nerv Syst. 2022 Jul 6;38(10):1885–94. doi: 10.1007/s00381-022-05590-9 (PMC9522746; doi:10.1007/s00381-022-05590-9)
Supplement: Supplementary file 1 — Supplementary file1 (DOCX 82 KB) [file 381_2022_5590_MOESM1_ESM.docx]

**Supplementary Material:**

Long-term quality of life after ETV in hydrocephalic pediatric patients

**Background**

The manuscript that has been created and uploaded includes all pediatric patients with at least one ETV. Often the medical history of the patiens is very complex, so vp shunt insertions or placement of external ventricle drains before or after ETV were performed. Retrospective evaluated, the significance of ETV is hard to identify. With focusing on patients that have only been treated with ETV in medical history a very small subgroup resulted. We therefore decided to examine all pediatric hydrocephalic patients with at least ETV treatment to gain insights about their quality of life.

In case of interest in study characteristics of the patients only treated with ETV we collected and evaluated these patients. Because of the small group we abstained publishing these results but would like to share the results for better understanding.

**Results**

**Study group characteristics**

By focusing patients only treated with ETV, 47 patients were treated also with vp shunt in further follow up and were excluded. 16 patients did not response, so 26 patients only with treatment by ETV answered the questionnaire. Patient’s characteristics are given in Table 1. Overview about the scheme of evaluation is given in Figure 1.


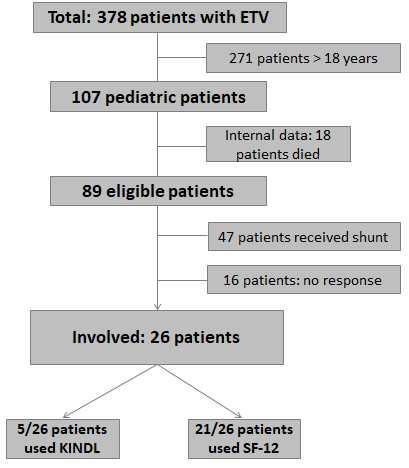


**Fig. 1** Flow chart about the response and the scheme of evaluation of patients treated only with ETV during 1993-2016.

| categorical variables | | n QoL study group (%) | n total study group (%) |
| --- | --- | --- | --- |
| total number |  | 26 | 107 |
| gender | male | 14 | 53 (49.5) |
|  | female | 12 | 54 (50.5) |
|  | ratio m:f | 1:0.86 | 1:1.02 |
| age at QoL evaluation (yrs) | mean ± SD | 22.7 ± 9.0 |  |
|  | < 6 | 1 |  |
|  | 6-14 | 4 |  |
|  | > 14 | 21 |  |
| age at time of ETV (yrs) | mean ± SD | 8,0 ± 5,9 | 6.9 ± 5.9 |
|  | < 6 | 9 | 53 (49.5) |
|  | 6-14 | 11 | 36 (33.6) |
|  | > 14 | 6 | 18 (16.8) |
| order of surgeries | ETV was first surgery | 18 | 65 (60.7) |
|  | surgeries before ETV | 8 | 42 (39.3) |
| etiologies | brain tumor | 10 | 40 (37.4) |
|  | brain abnormalitiy | 9 | 29 (27.1) |
|  | posthemorrhagic | 5 | 22 (20.6) |
|  | arachnoidal cyst | 1 | 3 (2.8) |
|  | postinfectious | 1 | 11 (10.3) |
|  | posttraumatic | 0 | 2 (1.9) |
| follow-up (years) ± SD |  | 12,2 | 9.9 ± 7.5 |

**Table 1** Patients’ characteristics of QoL-ETV-group and total study group. Statistical significance set at p<0.05. Statistical tests were done with Mann-Whitney U-test and Chi-square test.

**Quality of Life**

In general, 25 patients (96.2%) described their health status at least as “good”. Only 1 patient (3.8%) reported subjective health as “average”. None of our tests led to statistical significant differences between males and females, that’s why we show the following results without gender differentiation.

**Patients younger than 14 years**

5/26 patients (1f, 4m) received the KINDL-R questionnaire that was completed by their parents. Mean age at evaluation was 12.2 years. Mean age at ETV was 3.4 years. Noteworthy, 3/4 school-aged children attend a special school for handicapped children, only 1 child attends the middle school. The results of the KINDL-R are shown in Figure 2. All evaluated dimensions (body, psyche, self-esteem, family, friends, school and total score) are located below the corresponding scores of the reference group ^[18](#_CTVL00103fba933f1af4e9e9f529b94f0665edd)^. None of the dimensions showed a statistical significant difference.


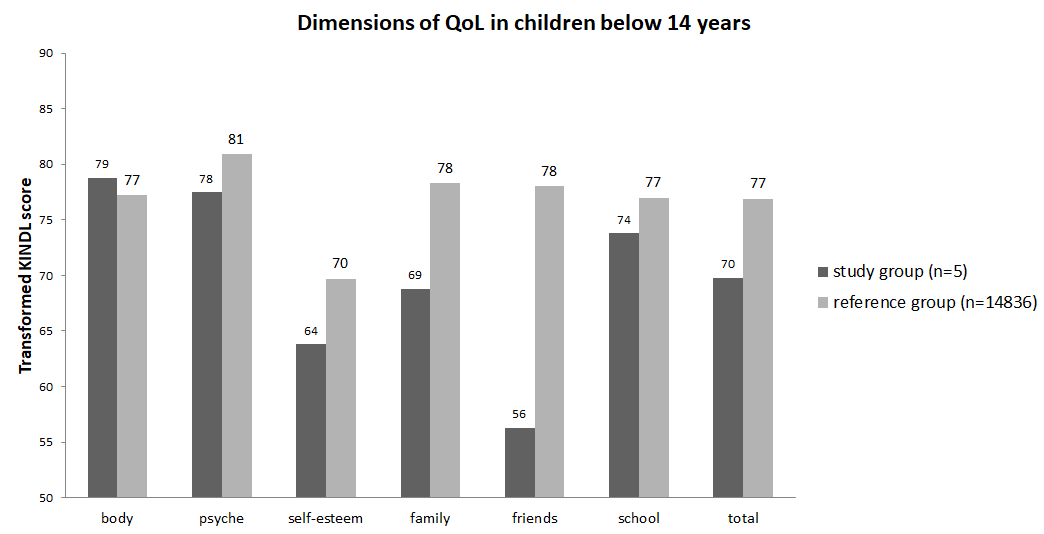


**Fig. 2** Average health dimensions evaluated with KINDL-R questionnaire in children. Statistical significance set at p<0.05. Statistical tests were done with Welch’s t-test.

|  | study group (n=5) | reference group (n=14836) | p value |
| --- | --- | --- | --- |
| body | 79 | 77 | 0.876 |
| psyche | 78 | 81 | 0.728 |
| self-esteem | 64 | 70 | 0.481 |
| family | 69 | 78 | 0.341 |
| friends | 56 | 78 | 0.070 |
| school | 74 | 77 | 0.551 |
| total | 70 | 77 | 0.298 |

**Table 2** Average health dimensions evaluated with KINDL-R questionnaire in children. Statistical significance set at p<0.05. Statistical tests were done with Welch’s t-test.

**Patients older than 14 years**

The SF-12 was completed by 21/26 patients (11f, 10m). The mean age of the survey responders was 22.7 years (±9.0 years). Mean age at time of operation was 8.0 years (±5.9 years). The physical dimension of the study group (PCS) reached a score of 48.9 vs. 49.0 of the reference group (p=0.883). The mental well-being of the study group shows a score of 50.5 vs. 52.2 of the reference group (p=0.834). So, neither the PCS nor the MCS of the observed study group show a significant lower result (see overview given in Figure 3).

Statistical values are given in Table 3; overview is given in Figure 3.


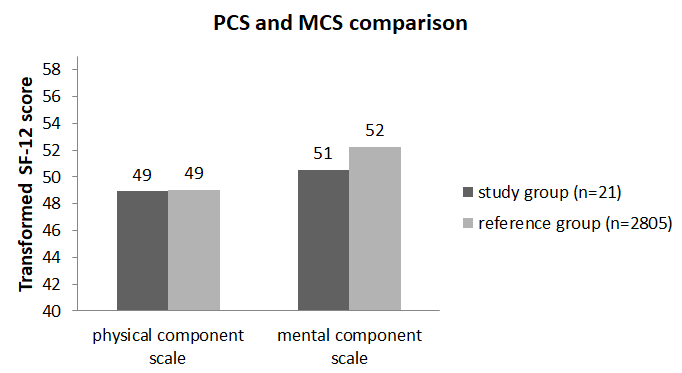


**Fig. 3** PCS and MCS of patients compared to reference group. Significance level set at p<0.05. Statistical analysis was done in pairwise comparisons of the ETV groups with the normal reference group using Mann-Whitney U-tests.

|  | study group | reference group | p value |
| --- | --- | --- | --- |
| physical component scale | 48.92 | 49.03 | 0.954 |
| mental component summary | 50.53 | 52.24 | 0.421 |

**Table 3** Comparisons between PCS and MCS of patients with ETV and reference group. Significance level set at p<0.05. Statistical analysis was done using Mann-Whitney U-tests.
